# Genetic Characterization of Coenzyme A Biosynthesis Reveals Essential Distinctive Functions during Malaria Parasite Development in Blood and Mosquito

**DOI:** 10.3389/fcimb.2017.00260

**Published:** 2017-06-20

**Authors:** Robert J. Hart, Amanah Abraham, Ahmed S. I. Aly

**Affiliations:** Department of Tropical Medicine, Tulane UniversityNew Orleans, LA, United States

**Keywords:** malaria, *Plasmodium*, blood, mosquito, Coenzyme A, vitamin B_5_

## Abstract

Coenzyme A (CoA) is an essential universal cofactor for all prokaryotic and eukaryotic cells. In nearly all non-photosynthetic cells, CoA biosynthesis depends on the uptake and phosphorylation of vitamin B5 (pantothenic acid or pantothenate). Recently, putative pantothenate transporter (PAT) and pantothenate kinases (PanKs) were functionally characterized in *P. yoelii*. PAT and PanKs were shown to be dispensable for blood stage development, but they were essential for mosquito stages development. Yet, little is known about the cellular functions of the other enzymes of the CoA biosynthesis pathway in malaria parasite life cycle stages. All enzymes of this pathway were targeted for deletion or deletion/complementation analyses by knockout/knock-in plasmid constructs to reveal their essential roles in *P. yoelii* life cycle stages. The intermediate enzymes PPCS (Phosphopantothenylcysteine Synthase), PPCDC (Phosphopantothenylcysteine Decarboxylase) were shown to be dispensable for asexual and sexual blood stage development, but they were essential for oocyst development and the production of sporozoites. However, the last two enzymes of this pathway, PPAT (Phosphopantetheine Adenylyltransferase) and DPCK (Dephospho-CoA Kinase), were essential for blood stage development. These results indicate alternative first substrate requirement for the malaria parasite, other than the canonical pantothenate, for the synthesis of CoA in the blood but not inside the mosquito midgut. Collectively, our data shows that CoA *de novo* biosynthesis is essential for both blood and mosquito stages, and thus validates the enzymes of this pathway as potential antimalarial targets.

## Introduction

CoA is an essential cofactor for all prokaryotes and eukaryotes to support a large number of metabolic processes including fatty acid biosynthesis and oxidation, as well as carbohydrate and amino acid metabolism (Lipmann et al., 1947a,b; Leonardi et al., 2005). In many living cells, the transport and utilization of pantothenic acid (vitamin B_5_, pantothenate in ionic form) is essential for CoA biosynthesis (Leonardi and Jackowski, 2007). The availability of several pantothenate analogs has enabled their usage in blocking the synthesis of CoA in bacteria, fungi and protozoa, thus emphasizing the importance of the pantothenate transport and phosphorylation as possible antimicrobial drug targets (Spry et al., 2008). However, recent data have shown that pantothenate analogs could also inhibit CoA biosynthesis in mammalian cells (Virga et al., 2006; Zhang et al., 2007).

Earlier studies in bird malaria parasites and recent studies in the human malaria parasite *P. falciparum* have shown that pantothenate analogs and pantothenamides inhibit intra-erythrocytic development of *P. falciparum* (Spry et al., 2005, 2010, 2013; de Villiers et al., 2017), and exert their antimalarial activity by possibly inhibiting pantothenate phosphorylation or the phosphorylation of alternative substrates (e.g., pantetheine) and thus blocking CoA biosynthesis. The cellular machinery for the biosynthesis of CoA from exogenous pantothenate involves a putative pantothenate transporter (PAT) and five enzymes, PanK (Pantothenate Kinase), PPCS (Phosphopantothenylcysteine Synthase), PPCDC (Phosphopantothenylcysteine Decarboxylase), PPAT (Phosphopantetheine Adenylyltransferase), and DPCK (Dephospho-CoA Kinase) (Leonardi et al., 2005; Spry et al., 2008). In most living organisms, PanK catalyzes the phosphorylation of pantothenate as an obligatory step in the canonical CoA biosynthesis pathway (Dansie et al., 2014). However, it was also shown that some pathogenic bacterial species, *Caenorhabditis elegans, Drosophila melanogaster*, and even human cells in culture rely on the uptake of pantetheine inside cells and use the PanK to generate 4′ phosphopantetheine (Jackowski and Rock, 1984; Leonardi et al., 2005, 2010; Zhang et al., 2007; Spry et al., 2008; Sibon and Strauss, 2016). In this case, 4′ phosphopantetheine is further processed by PPAT and DPCK to produce CoA without the intermediate canonical cysteine addition and cysteine decarboxylation steps.

We have previously investigated the role of the malaria parasite putative PAT and two candidate PanK genes of *P. yoelii* in the development of this malaria parasite in murine erythrocytes and *Anopheles* mosquitoes (Hart et al., 2014, 2016a; Kehrer et al., 2016). Our studies that involved gene disruption and expression analyses demonstrated that the PAT, PanK1, and PanK2 genes are dispensable for blood stage development in mice but are crucial for oocyst development and sporozoite formation in the mosquito. Herein, we are investigating the role of the other four CoA biosynthesis enzymes in the development of *P. yoelii* in mouse and mosquito models.

## Results

### Conservation of the CoA biosynthesis enzymes in all *Plasmodium* species

Although, CoA biosynthesis inhibition has been studied in *Plasmodium*, the genes encoding this pathway have not been biochemically characterized in any malaria parasite species. The availability of genome sequence data from different species of protozoan parasites made it possible to search for homologs of CoA biosynthesis enzymes in the *Plasmodium* genome database. All the eukaryotic–type enzymes of CoA de novo biosynthesis pathway are conserved in all *Plasmodium* species (http://mpmp.huji.ac.il/maps/coasynthesis.html).

**Figure 1:**
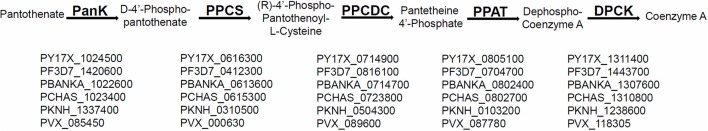
Conservation of CoA biosynthesis pathway in all malaria parasite species. A schematic representation of the canonical biosynthesis pathway of CoA from pantothenate with enzymes PanK (Pantothenate Kinase), PPCS (Phosphopantothenylcysteine Synthase), PPCDC (Phosphopantothenylcysteine Decarboxylase), PPAT (Phosphopantetheine Adenylyltransferase), and DPCK (Dephospho-CoA Kinase), respectively. The PlasmoDB IDs for the genes encoding the biosynthesis enzymes of CoA, that are conserved by homology and synteny in all sequenced malaria parasite species, are listed under each enzyme in *P. yoelii, P. falciparum, P. berghei, P. chabaudi, P. knowlesi*, and *P. vivax*, respectively.

The *P. yoelii* rodent malaria parasite orthologs for the CoA biosynthesis enzymes PPCS (PlasmoDB ID: PY17X_0616300), PPCDC (PlasmoDB ID: PY17X_0714900), PPAT (PlasmoDB ID: PY17X_0805100), and DPCK (PlasmoDB ID: PY17X_1311400) are all highly conserved by chromosomal synteny and share considerable amino acid identity with other *Plasmodium* species orthologs (Figure 1). It should be noted that, unlike mammalian cells and similar to bacteria, the PPAT and DPCK enzymes are encoded on two different genes and not on one gene that encodes a bifunctional protein.

### Parasites lacking PPCS and PPCDC display normal blood stages growth rates

To determine the role of the intermediate enzymes of the canonical pantothenate CoA biosynthesis (PPCS and PPCDC) in the development of parasite within host erythrocytes and in transmission to the mosquito, a genetic approach was employed to generate individual deletion of PPCS and PPCDC in *P. yoelii* 17 X-NL non-lethal strain (*Py*WT). To create individual gene deletion of *Py*PPCS and *Py*PPCDC, a targeting vector, pAA20, containing the enhanced GFP (eGFP) reporter cassette and the human DHFR resistance selection marker cassette was used to clone 5′ and 3′ regions of each gene. Following transfection with the targeting vectors, drug selection and cloning of transgenic parasites, genomic PCR analyses were performed to confirm the genetic replacement events and the deletion of *Py*PPCS and *Py*PPCDC (Supplementary Figure 1).

**Figure 2:**
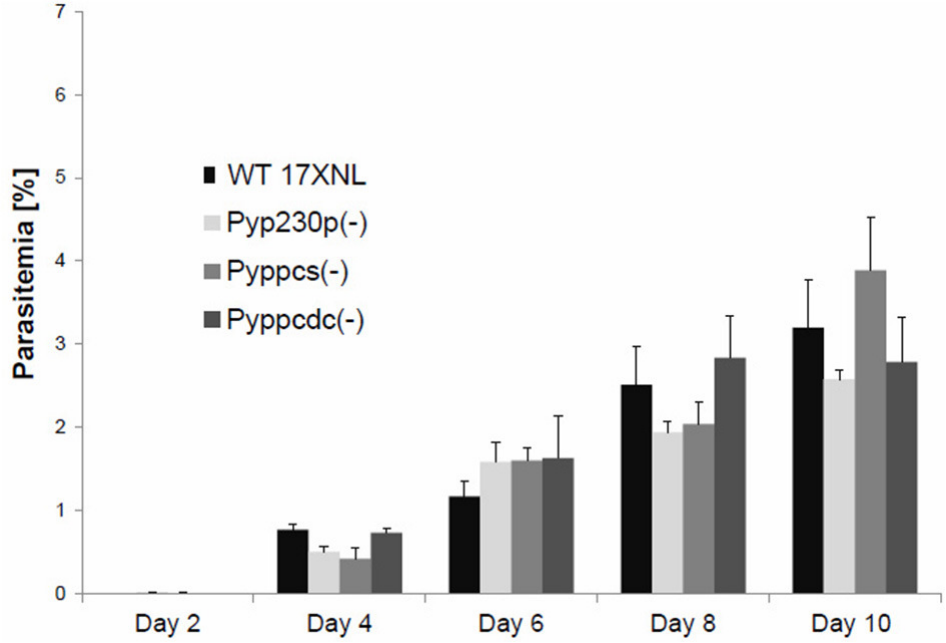
Targeted deletion of PPPCS or PPCDC in *P. yoelii* has no effect on blood stages development. Graph shows the average blood stage parasitemia (% infected erythrocytes out of >5,000 cells counted) in groups of four BALB/c mice per strain following IV injection of 5000 infected erythrocytes. The mean values for all parasite strains were analyzed with the One-Way Analysis-of-Variance (ANOVA) and statistical significance was set at a *P* < 0.05. This experiment confirmed that the deletion of *PyPPCS* or *PyPPCDC* does not lead to any statistical significant reduction in blood stage parasitemia compared to WT and WT-like *Pyp230p*(−) parasites.

To assess the effect of *Py*PPCS and *Py*PPCDC deletion during the intraerythrocytic phase of *P. yoelii* development, we compared blood stage (BS) parasitemia of groups of four BALB/c mice per genotype intravenously (IV) infected with 5,000 BS of *Pyppcs(*−*)* clone Q4, *Pyppcdc(*−*)* clone R3, WT-like parasites *Pyp230p(*−*)* clone A5 (Hart et al., 2014, 2016a,b) and 17 X-NL WT parasites (Figure 2). Parasitemia was recorded daily from giemsa-stained thin blood smears until clearance at day 17 or 18 post-infection (PI). The mean values for all parasite strains were analyzed with the One-Way Analysis-of-Variance (ANOVA) and statistical significance was set at a *P* < 0.05. No significant differences were detected on any of the tested days between any of the different genotypes (Figure 2). These findings demonstrate that the deletion of both PPCS and PPCDC do not affect the development of blood stage parasites.

### *Py*PPCS and *Py*PPCDC are not critical for development of *P. yoelii* blood sexual stages

**Figure 3:**
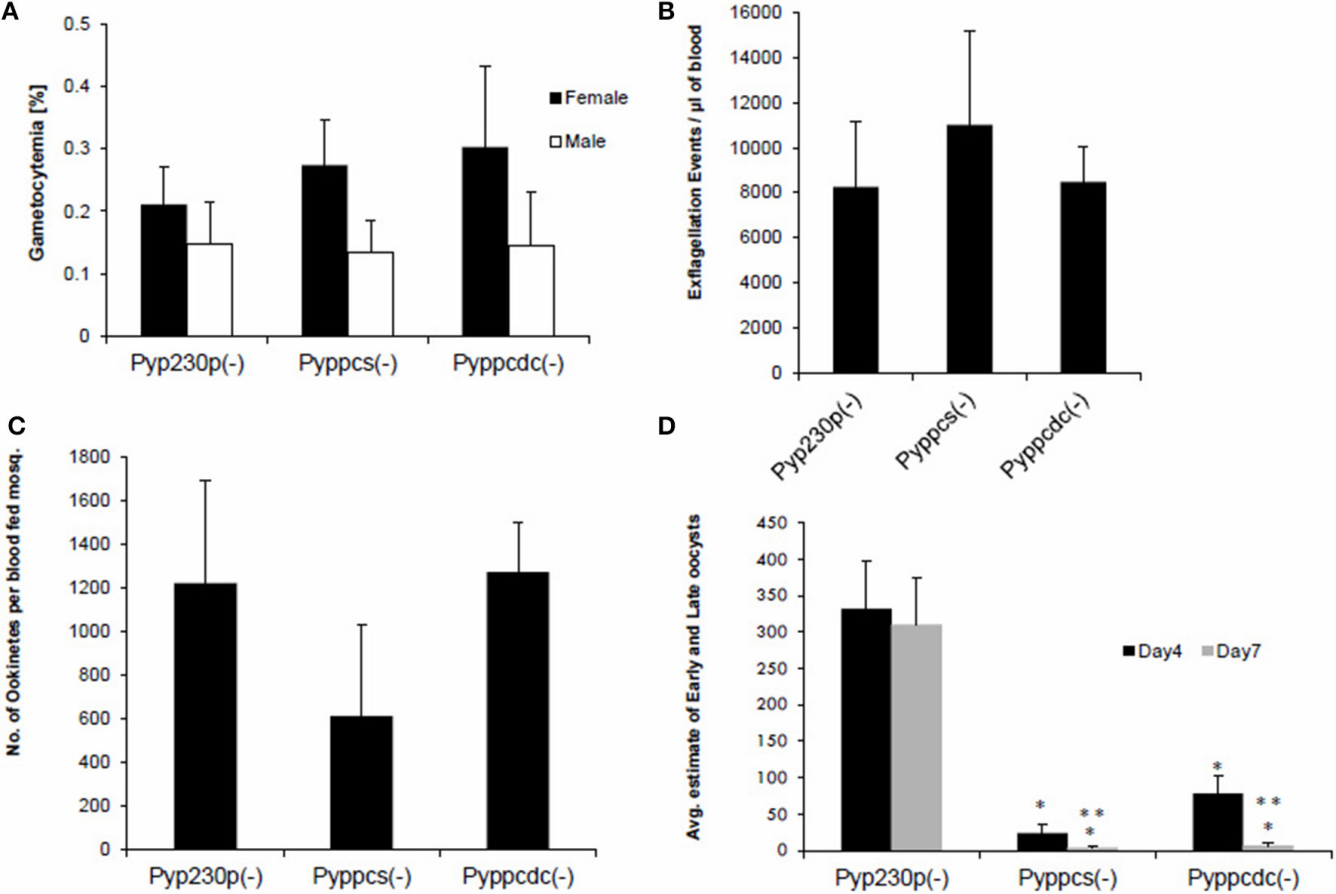
Parasites lacking *PyPPCS* or *PyPPCDC* are deficient in oocyst development. **(A)** Graph shows average percentages of male and female mature gametocytes in thin smears from groups of SW mice IV injected 3 days earlier with 10^6^ infected erythrocytes with *Pyppcs(*−*), Pyppcdc(*−*)*, or *Pyp230p*(−) (all are G5 passages). No statistical significant difference is detected among strains tested in sexual stages development in blood. **(B)** Graph shows average number of male gamete exflagellation events per μl of mouse blood determined by a hemocytometer using 1:10 dilution of tail blood. No statistical significant difference in male gamete exflagellation is detected in *Pyppcs(*−*)* or *Pyppcdc(*−*)* strains compared to *Pyp230p*(−). **(C)** Graph shows average number of ookinetes per mosquito dissected out 20 hours pmf with no statistical significant difference among strains tested. Results in graphs **(A–C)** confirm that the development of sexual stages and ookinetes is not affected by the deletion of *PPCS* or *PPCDC*. **(D)** Graph shows the average number of early oocysts developing on midguts of mosquitoes infected with *Pyppcs(*−*), Pyppcdc(*−*)*, or *Pyp230p*(−) strains at days 4 and 7 pmf detected using fluorescence microscopy. Statistical significant reduction (denoted by ^*^) in the number of developing oocysts is detected in *Pyppcs(*−*)* or *Pyppcdc(*−*)* strains compared to *Pyp230p*(−) at days 4 and 7 pmf, respectively. Further statistical significant reduction (denoted by ^**^) in the number of developing oocysts at day 7 compared to day 4 pmf is detected in each of the strains *Pyppcs(*−*)* and *Pyppcdc(*−*)* but not in *Pyp230p*(−) strain. The mean values for all parasite strains were analyzed with the One-Way Analysis-of-Variance (ANOVA) and statistical significance was set at a *P* < 0.05. The results shown are the mean of three independent experiments with each independent experiment involving at least three mice per parasite strain, error bars represent standard deviation.

The viability of *Pyppcs(*−*)* and *Pyppcdc(*−*)* parasites in mouse erythrocytes made it possible to assess the function of both *Py*PPCS and *Py*PPCDC in gametocyte development and maturation, as well as in gametogenesis. Outbred Swiss Webster (SW) mice were intravenously (IV) infected with 1 × 10^6^ BS of *Pyppcs*(−), *Pyppcdc(*−*)*, or *Pyp230p(*−*)* parasites. Blood samples were collected and smears were made simultaneously at day three post infection to determine the percentages of male and female gametocytes in giemsa-stained thin blood smears (Figure 3A), and male gamete exflagellation rate for the three strains (Figure 3B). Similarly, the average of male and female gametocytmeias were not significantly different between the different genotypes. Male gamete exflagellation events were also not significantly different between the four strains (*P*-value of 0.88). These experiments confirm that the lack of the second and third enzymes of CoA biosynthesis PPCS and PPCDC do not interfere with the growth and development of any asexual or sexual blood stage parasites.

### Parasites lacking *PPCS and PPCDC* produce ookinete but fail to produce mature oocysts and oocyst sporozoites

**Figure 4:**
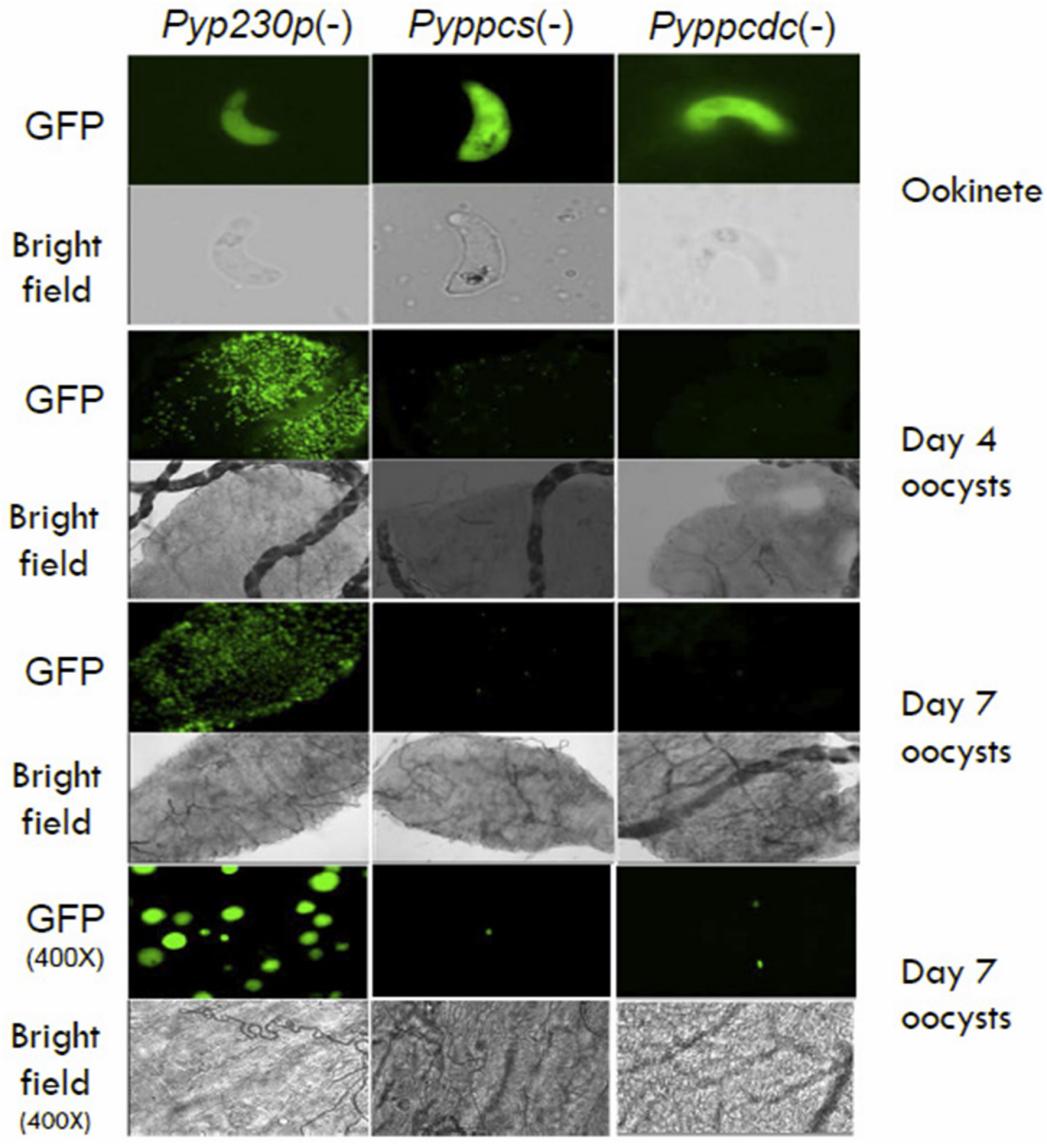
The morphology of *Pyppcs(*−*)* and *Pyppcdc(*−*)* oocysts appear diminished in size. Fluorescence and bright field microscopy images of ookinetes 20 hours pmf (400X magnification), oocysts at day 4 pmf (100X magnification), oocysts at day 7 pmf (100X magnification), and oocysts at day 7 pmf (400X magnification) dissected out of mosquitoes infected with *Pyppcs(*−*), Pyppcdc(*−*)*, or *Pyp230p*(−) strains. Images show the morphology of *Pyppcs(*−*)* and *Pyppcdc(-)* oocysts appear diminished in size and fluorescence compared to the oocysts of the oocysts of *Pyp230p*(−), but the ookinetes of all three strains appear normal in size and morphology.

To examine the importance of *PPCS* and *PPCDC* for the development of ookinetes, female *Anopheles stephensi* mosquitoes were fed on infected mice displaying the highest male gamete exflagellation rate from the previous experiment. After 20 h post mosquito-feeding (pmf), mosquito midguts from at least 20 blood-fed female *A. stephensi* were dissected, and mature ookinetes were counted for each parasite genotype (Figure 3C). Despite an apparent slight reduction in the average number of ookinete in the knockout parasites, no statistical significant difference was detected compared to the control strain. These data suggest so far that *Py*PPCS and *Py*PPCDC do not play essential roles in fertilization, zygote formation, and ookinete maturation inside the mosquito. To investigate the importance of *Py*PPCS and *Py*PPCDC in oocyst formation and sporozoite development, we took advantage of the fact that *Pyppcs(*−*), Pyppcdc(*−*)*, and *Pyp230p(*−*)* parasites were all designed to constitutively express eGFP in all life cycle stages to visualize the development of early oocyst. Midguts of mosquitoes fed on *Pyppcs(*−*), Pyppcdc(*−*)*, and *Pyp230p(*−*)* parasites were dissected, and the number of oocysts on their midguts were counted using fluorescence microscopy at day 4 and 7 pmf, respectively (Figure 3D). Results from three independent experiments revealed a significant reduction in the number of early oocysts for both knockout parasites compared to *Pyp230p(*−*)* at day 4 pmf. More significant reduction at day 7 pmf was displayed by both knockout strains compared to the control strain at day 7 pmf, and to the respective knockout strains at day 4 (Figure 3D). Furthermore, the fluorescence displayed the oocysts of the knockout strains were barely detectable and the size of the knockout oocysts was considerably diminished compared to the control strain oocysts (Figure 4). More importantly, no oocyst sporozoites could be detected at day 10 pmf in mosquitoes fed on *Pyppcs(*−*) or Pyppcdc(*−*)* infected mice in four independent experiments (Table 1). Altogether, this data demonstrates that the lack of PPCS or PPCDC completely blocked oocyst and sporozoite development. These observations are in agreement with our previous results about the essential roles of PAT, PanK1, and PanK2 in mosquito stages development (Hart et al., 2014, 2016a). This highlight the essential need of pantothenate acquisition, phosphorylation and utilization in *de novo* CoA biosynthesis in *Plasmodium* development in the mosquito vector, with an alternative route or mechanism for CoA biosynthesis in the blood.

**Table 1:** Oocyst Sporozoite formation is completely abolished in *Pyppcs(*−*)* and *Pyppcdc(*−*)*.

**Average Number of Midgut Oocyst Sporozoites per Mosquito (Day 10 pmf)**			
	***Pyppcs(**−**)***	***Pyppcdc(**−**)***	***Pyp230p(**−**)***
Experiment 1	0	0	8,800
Experiment 2	0	0	5,172
Experiment 3	0	0	7,886
Experiment 4	0	0	6,250

### PPAT and DPCK are essential for growth of blood stage parasites

**Figure 5:**
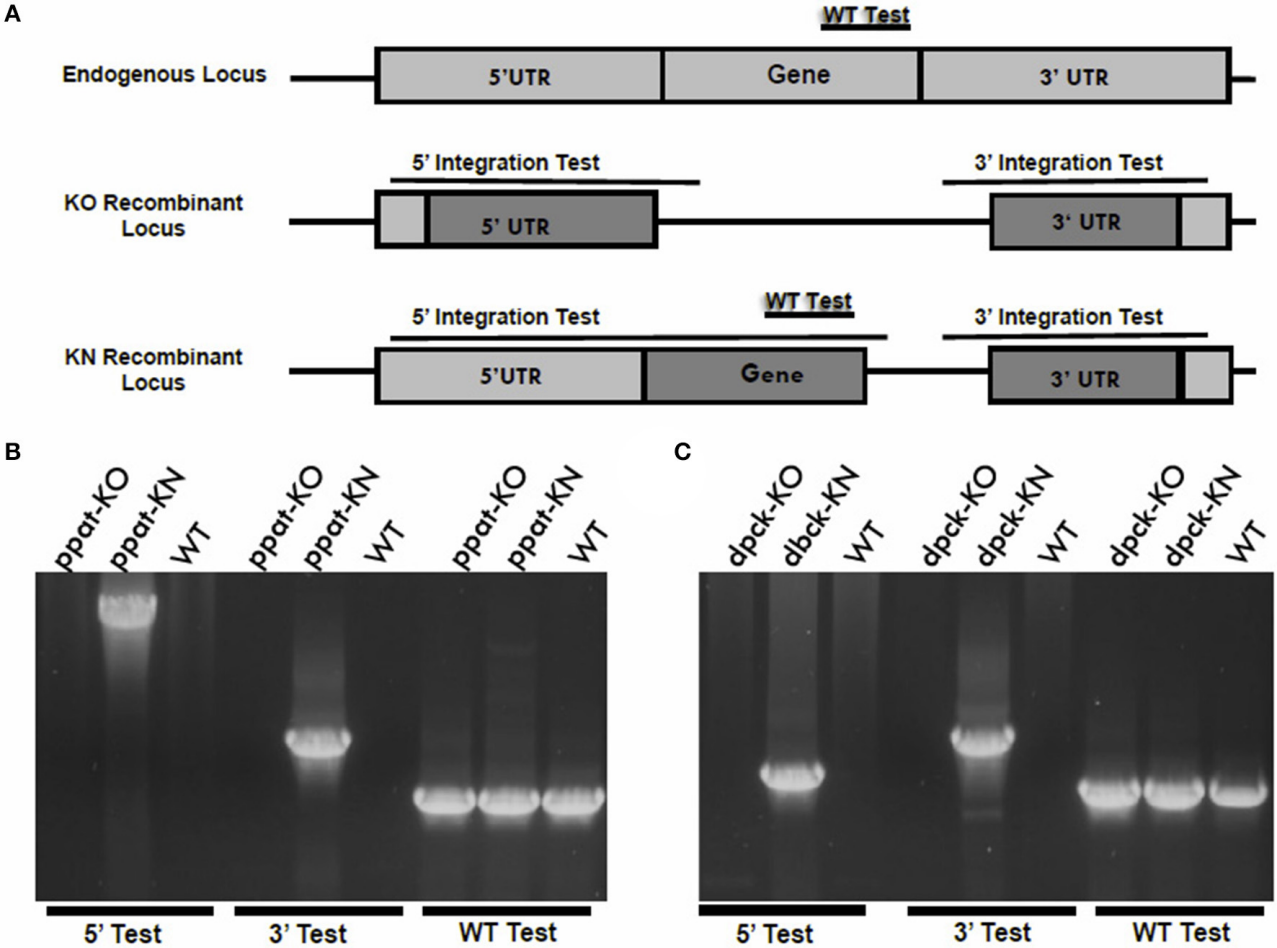
Knockout vs. knock-in gene targeting analyses reveal essential functions of PPAT and DPCK during blood stages development. **(A)** A schematic representation of the knockout vs. knock-in gene targeting strategy in which an endogenous gene locus (light gray boxes) is targeted for deletion or complementation by knockout (KO) and knock-in (KN) plasmid constructs, respectively. The knockout and knock-in plasmid constructs (dark gray boxes) share the same 3′ fragment but have different 5′ fragments that either disrupts or complement, respectively, the coding sequence of a gene by homologous double cross-over recombination. If the gene is essential for the functions of the parasite blood stages, no KO parasites will be recovered following transfection. If the gene is accessible for genetic modification, the KN parasites will be recovered. Diagnostic WT-specific or integration-specific test amplicons are indicated by lines. 36-cycles PCR genotyping confirmed the integration of KN but the KO plasmid constructs into PPAT gene locus **(B)** and into DPCK gene locus **(C)**. Integration of ppat-KN and dpck-KN plasmid constructs were confirmed using oligonucleotide primer combinations that can only amplify from the recombinant loci (5′ Test and 3′ Test) in **(B,C)**, respectively. The WT-specific PCR reaction (WT) confirmed that the parasites recovered from transfection with ppat-KO **(B)** and dpck-KN **(C)** were resistant WT parasites.

To determine the roles of the last two enzymes of pantothenate CoA biosynthesis, PPAT and DPCK, in parasite development in host erythrocytes, we employed the same genetic targeting approach to generate single gene deletions of PPAT and DPCK in *P. yoelii* 17X-NL parasites. The first attempts to delete the genes were not successful. In order to distinguish between a technical or genetic inability to delete the genes, we employed a knockout/knock-in gene targeting technique. In these experiments, the knock-in plasmid construct targets the gene to replace part or the whole coding sequence with its identical copy followed by a constitutive 3′ UTR (Figure 5A). The knockout plasmid construct shares the same 3′ region of the targeted sequence for the homologous recombination, while the 5′ region will be used to delete the gene or replace it with its own copy by the knockout and knock-in constructs, respectively (Figure 5A). The gene targeting experiments with both constructs for each gene are conducted during the same transfection experiment. Following transfection with the targeting vectors, drug selection and cloning of transgenic parasites, genomic PCR analyses were performed to examine the integration of the targeting constructs for PPAT and DPCK. Only the knock-in targeting constructs were integrated in the chromosomal loci for PPAT (Figure 5B) and DPCK (Figure 5C), respectively, but the knockout constructs of each didn't integrate. These results confirm that the inability to delete *PyPPAT* and *PyDPCK* is not due to a technical reason, nor the inability to access the gene loci, but due to a crucial role that both enzymes play in the growth and/or survival of blood stage parasites in mouse erythrocytes. Thus, and in contrast to the first two enzymes of this pathway, the last two enzyme for CoA biosynthesis are essential for blood stage parasites. This indicates that *de novo* CoA biosynthesis indeed occurs in blood stage, however, by unconventional first and intermediate pathway events.

## Discussion

**Figure 6:**
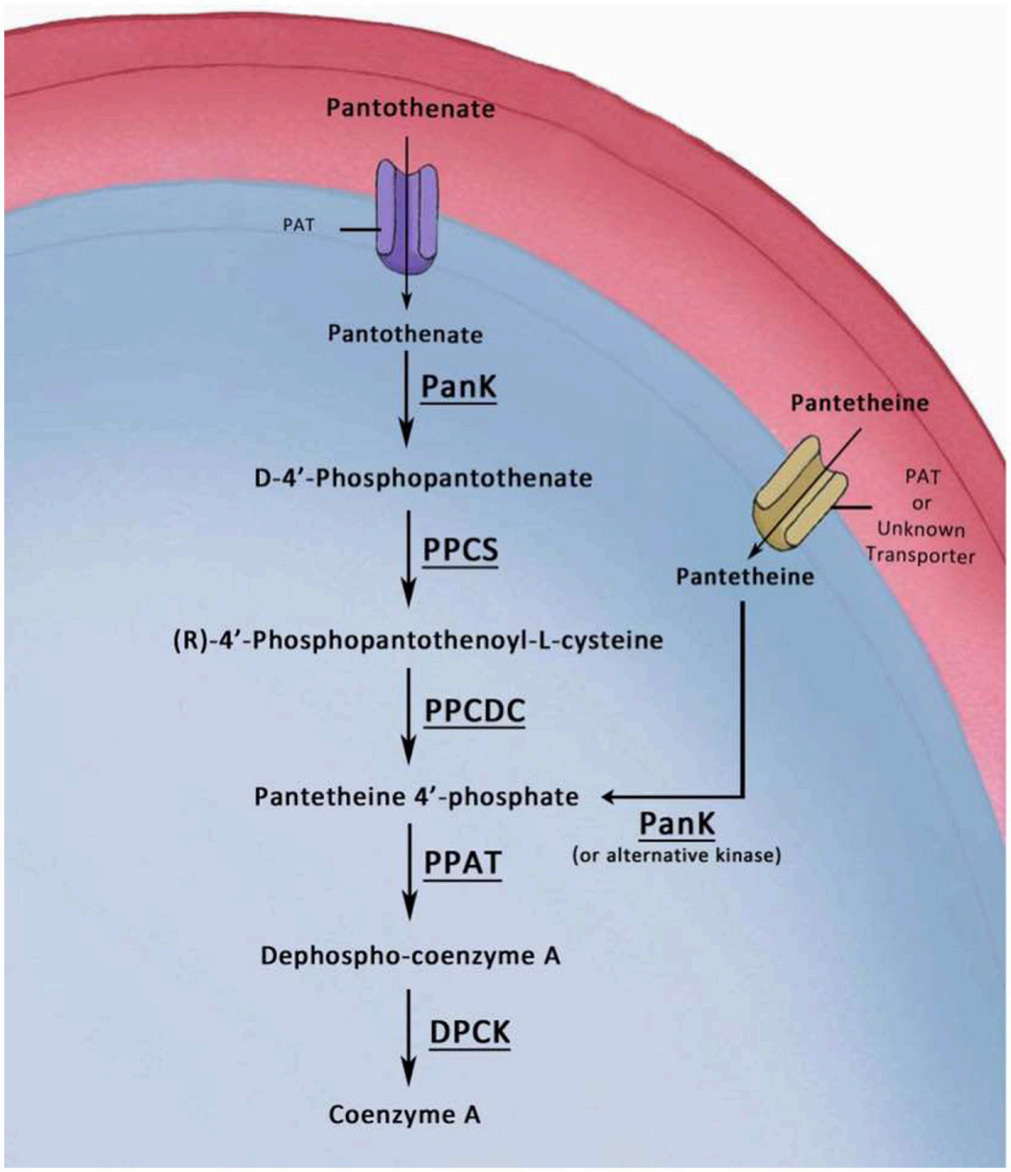
Model of the Alternative CoA biosynthesis pathways in *P. yoelii* blood stages. In this graphic model, pantothenate is transported into the parasite by a pantothenate transporter (PAT). The first enzymatic step in CoA biosynthesis is the phosphorylation of pantothenate by a pantothenate kinase (PanK) to form 4′-phosphopantothenate (PPA). Addition of cysteine to 4′-PPA by a Phosphopantothenylcysteine Synthase (PPCS) produces 4′-phospho-N-pantothenolycysteine (PPC). Decarboxylation of PPC by a PPC decarboxylase (PPCDC) produces 4′-phosphopantetheine (PP). Adenylation of PP by Phosphopantetheine Adenylyltransferase (PPAT) produces Dephospho-CoA. The latter is phosphorylated by a Dephospho-CoA Kinase (DPCK) to produce CoA. The other alternative depicted route of CoA biosynthesis from pantetheine has been confirmed in prokaryotes and eukaryotes. Transport of pantetheine could be accomplished by PAT or by another unidentified transporter. Pantetheine can be phosphorylated inside the parasite by PanK (or alternative kinase) to 4′ phosphopantetheine, which is the substrate for PPAT, and thus bypassing the PPCS and PPCDC enzymes.

Historically, the main efforts in researching the utilization and processing of pantothenic acid have focused solely on the intracellular malaria parasite blood stages. We explored the cellular roles of the putative enzymes that are implicated in CoA biosynthesis, first with PAT and next with PanK1 and PanK2 (Hart et al., 2014, 2016a). It was concluded that PAT, PanK1, and PanK2 are not needed during development of asexual or sexual blood stages, but are essential for the establishment and completion of mosquito stages development. Furthermore, our results here show that PPCS and PPCDC have critical roles for the development of mosquito stages but are dispensable for blood stage development. In light of earlier results that *P. lophurae* blood stages can salvage CoA from the host nucleated RBCs (Brohn and Trager, 1975; Trager and Brohn, 1975), these initial results could indicate that *P. yoelii* is utilizing the same unknown mechanism to salvage CoA from mouse host erythrocytes. However, our data here also shows that PPAT and DPCK, which are the last enzymes involved in *de novo* CoA biosynthesis, are essential for the survival and growth of blood stages. Importantly, earlier studies also confirmed that *P. lophurae* could salvage CoA biosynthesis intermediates for the survival of blood stage parasite in the absence of CoA in the medium (Brohn and Trager, 1975; Trager and Brohn, 1975). These intermediates were strictly the 4′ phosphopantetheine (the substrate of PPAT) and Dephospho Coenzyme A (the substrate of DPCK), but not the substrates of PPCS or PPCDC. It was speculated by Trager and Brohn that the host PPAT and DPCK inside the nucleated erythrocytes are responsible for the conversion of the last two intermediates to CoA, which in return can be salvaged by the intracellular parasite, or *P. lophurae* could have the enzymes (Brohn and Trager, 1975; Trager and Brohn, 1975). However, the nucleated metabolically active avian erythrocytes are different from mouse or human erythrocytes, and there is no evidence or indication that the mammalian bi-functional PPAT-DPCK protein or mouse CoA are present in mature mouse erythrocytes. Importantly, it was recently shown in *C. elegans* and *D. melanogaster* and human cell in culture that CoA biosynthesis could be synthesized via phosphorylated CoA intermediates other than pantothenate (de Villiers and Strauss, 2015; Sibon and Strauss, 2016). Thus, our results indicate malaria parasite blood stages uses alternative mechanism for *de novo* CoA synthesis, with obligatory roles for the last two biosynthesis enzymes which are PPAT and DPCK (Figure 6).

The alternative pathway for CoA biosynthesis was first identified in pathogenic prokaryotes, and later in several eukaryotic systems, where pantetheine is used as a substrate instead of pantothenate and this in turn bypasses the cysteine addition and carboxylation steps by PPCS and PPCDC enzymes, respectively (Jackowski and Rock, 1984; Leonardi et al., 2005, 2010; Zhang et al., 2007; Spry et al., 2008; Sibon and Strauss, 2016). Based on our results, we proposes a model for the biosynthesis of CoA in blood stages in which pantetheine is transported, by PAT or another yet unknown transporter, to the parasite where there will be no need for PPCS and PPCDC in CoA biosynthesis (Figure 6). Importantly, the initial extracellular mosquito stages (gametes, zygotes, and ookinetes) were shown to be unaffected by the lack of the canonical CoA pathway, most probably due to the same mechanism that rescued their development in mouse blood, which could be also in theory be utilized in mosquito midguts in the first 24 h following transmission (Aly et al., 2009). However, there are no evidence for alternative routes of CoA acquisition or biosynthesis, other than the canonical CoA biosynthesis from pantothenate, utilized by the extracellular oocysts developing on mosquito midguts.

According to our model and if pantetheine is transported into the parasite (Figure 6), it must be phosphorylated to become 4′ phosphopantetheine, which is the substrate for PPAT. This can be accomplished most probably by one of the *Plasmodium*-encoded PanKs. Nevertheless, other unknown kinases can also contribute to this function as a recent study indicates that both PanKs can be simultaneously deleted from blood stages with no apparent significant effect on asexual and sexual blood stage development (Srivastava et al., 2016). Importantly, CoA pathway has been historically and is still recently considered as a potential target for the development of antimalarial chemotherapeutic agents against the pathogenic blood stages (Spry et al., 2005, 2008, 2010, 2013). The results presented here provide solid evidence for the *de novo* biosynthesis of CoA by rodent malaria parasites during the blood stage cycle. In addition, it further confirms the antimalarial transmission potential of CoA biosynthesis inhibitors. It will be interesting to explore the potential prophylactic effect of CoA biosynthesis inhibitors on the malaria parasite pre-erythrocytic stages.

## Materials and methods

### Experimental animals, parasites, and mosquitoes

Female Swiss Webster (SW) mice and BALB/c mice (6–8 weeks old) were purchased from Envigo Research Models and Services, formerly known as Harlan Laboratories, (Indianapolis, Indiana). All Animal experiments were conducted according to the approved protocols of the Institutional Animal Care and Use Committee (IACUC) of Tulane University. All other experimental protocols and the use of recombinant DNA were conducted according to the approved protocols of the Institutional Biosafety Committee (IBC) of Tulane University. Detailed phenotypic analyses in mice and mosquitoes were performed, using *Pyp230p(*−*)* clone A5, *Pyppcs(*−*)*, clone Q4 and *Pyppcdc(*−*)* clone R3 parasites, as previously described (Aly et al., 2010; Hart et al., 2014, 2016a,b).

### Generation of transgenic parasites

Targeted gene deletions induced by homologous recombination were performed as previously described (Aly et al., 2010; Hart et al., 2014, 2016b). Briefly, 5′ and 3′ UTR regions of the target genes were amplified from genomic DNA of *P. yoelii* 17X-NL strain using primer pairs 71–72 and 73–74 for PPCS, respectively, and primer pairs 81–82 and 83–84 for PPCDC, respectively, and primer pairs 91–92 and 93–94 for PPAT, respectively, and primer pairs 101–102 and 103–104 for DPCK, respectively. Primer sequences are listed in Supplementary Table 1. The amplified fragments were inserted into the transfection plasmid pAA20 between *SacII* and *BamHI*, and *KpnI* and *HindIII*, respectively. For the knock-in constructs of PPAT and DPCK enzymes, the 5′ UTR region cloned in with *SacII* and *BamHI* and used for the homologous recombination in the knockout constructs were replaced by the coding sequence of PPAT and DPCK, amplified by primers 97–98 and 107–108, respectively. All the resulting targeting vectors were linearized with *SacII* and *KpnI* prior to transfection of *P. yoelii* 17X-NL parasites by electroporation with Lonza Nucleofector II. Selection of resistant parasites and cloning of transfectants were performed as previously described (Janse et al., 2006; Philip et al., 2013). To confirm the disruption of the targeted genes, gDNA was extracted from parasites clone and diagnostic PCR analyses were performed using primers pairs that test the 5′ integration, 3′ integration and to test for WT coding sequence (75–16, 17–76, and 77–78 for PPCS, respectively; 85–16, 17–86, and 87–88 for PPCDC, respectively; 95–16, 17–96, and 97–98 for PPAT, respectively; and 105–16, 17–106, and 107–108 for DPCK, respectively). Primer sequences listed in Supplementary Table 1.

### Statistical analyses

One-way Analysis-of-Variance (ANOVA) was used to assess the mean values for statistically significant differences in all experiments. GraphPad InStat software was used for all statistical analyses. *P* < 0.05 were considered statistically significant. Standard Deviation (SD) values were used to represent error bars in all graph figures.

## Author contributions

ASIA designed the experiments; RH and AA conducted the experiments; RH, AA, and ASIA analyzed the results; RH, AA, and ASIA wrote the manuscript.

### Conflict of interest statement

The authors declare that the research was conducted in the absence of any commercial or financial relationships that could be construed as a potential conflict of interest.
