# Supplementary material for: Genetic Characterization of Coenzyme A Biosynthesis Reveals Essential Distinctive Functions during Malaria Parasite Development in Blood and Mosquito
Source: Front Cell Infect Microbiol. 2017 Jun 20;7:260. doi: 10.3389/fcimb.2017.00260 (PMC5476742; doi:10.3389/fcimb.2017.00260)
Supplement: Supplementary file 1 [file DataSheet1.PDF]

Supplementary Online Data

Genetic characterization of Coenzyme A biosynthesis reveals essential distinctive functions during malaria parasite development in blood and mosquito.

Robert J. Hart, Amanah Abraham and Ahmed S. I. Aly*

Tulane University, Department of Tropical Medicine, New Orleans, LA 70112, USA.

***To whom correspondence should be addressed: **Ahmed S. I. Aly**, Tulane University, Department of Tropical Medicine, New Orleans, LA 70112, USA. Phone: +1-504-988-2286,

Email: aaly@tulane.edu

Supplementary Figure 1: Targeted deletion of PPCS and PPCDC.

A Schematic representations of the targeted deletion of (**A**) *Pyppcs*(-) parasites and (**C**) *Pyppcdc*(-) parasites. The endogenous *Py*PPCS and *Py*PPCDC genomic loci were targeted with replacement fragments containing the 5′ and 3′ *Py*PPCS and *Py*PPCDC UTRs flanking the human DHFR positive selection marker and eGFP cassettes. The two constructs were transfected during the same experiment. Diagnostic WT-specific or integration-specific test amplicons are indicated by lines. 36-cycles PCR genotyping confirmed the integration of gene-replacement construct using oligonucleotide primer combinations that can only amplify from the recombinant loci (5′ Test and 3′ Test) in (**B**) *Pyppcs*(-) and in (**D**) *Pyppcdc*(-). The WT-specific PCR reaction (WT) confirmed the absence of WT parasites in *Pyppcs*(-) and *Pyppcdc*(-) in (**B**) and (**D**), respectively.

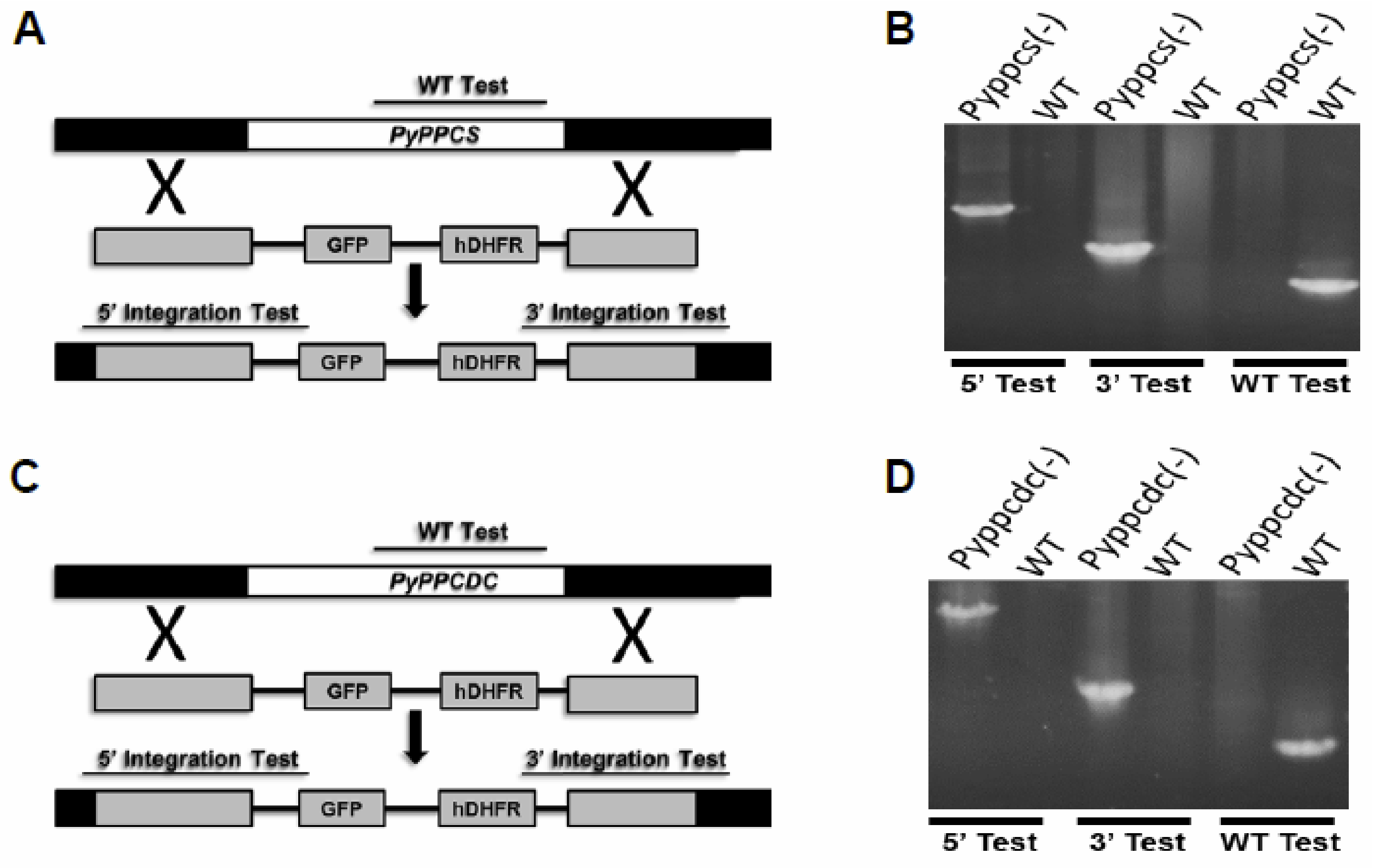

Supplementary Table 1

Primer sequences (5’-3’)

71: GGCCGCGGGCTGGAGACAAAATTATTGTTTTAGTCCAAATGCG;
72: TCCGGATCCTTGTTCATTTTCATTTTCATTATTTTGTTAATTTTC;
73: TGCCAAGCTTCAACTAAAAAAATGAATATATATTTATGAGCAA;
74: TCCGGTACCTCCTAAAATCACAGCAAATTTTGGCAAACAAAAT;
75: GAAGTTCCGACTATCAATTCGTTTCTCTACTTG;
76: AATTCGCAAATTTGCTATGTATGTATAAAGCCC;
77: GGCCGCGGTGTTAAGGAGAAATTCTCAAAAAGCTACAAAAA;
78: TCCGAATTCTTTCCATAATTTTCTATCAATCAAATGATTATA;
81: GGCCGCGGTCGATGAATATGTAAAGTGAATTATACAAAAAA;
82: TCCGGATCCCCATTCATCTTTATCTAATAGCACTTTTTCTTAAAA;
83: TGCATCGATTATCTTACCCTTTTTAATACTTCTTTTGTTTCAC;
84: TCCGGTACCCCAGAGAAAAATGCTGAAAATGTTGAAAATGCTG;
85: GTTGGGAAGGTTTCTCGAAGATTACGAGCAAGTA;
86: TGACTTGTGGATACACAACATGTGTGTGCAACCG;
87: GGCCGCGGTGTAGCTACAAATGTCGCATTTGAAAAATTTCTG;
88: TCCGAATTCTAACGTTTTCATGCATTCCATTATCTCTTTAATTAC;
91: GGCCGCGGGGATACCCTCTAAGTAGGTACATATACATGCTG;
92: TCCGGATCCTTAAAATCCATTTTCGTAAATCCCCCTATTCTTCAT;
93: TGCCAAGCTTGCGACAAAAAAATTATTATGATCCGTTTTATCCA;
94: TCCGGTACCTACATATTCCATTTGGCCGTATGGTGTGTGCGT;
95: TGTAATAAAAAGGGAAATCGAACTAATTAAGAA;
96: TAACATGGGGAAGCCCGATGATACTACTGTCAT;
97: GGCCGCGGAACATGCTTCCTTATCAATATTGCAAATGTGTA;
98: TCCGAATTCGAATAAATTTAAATGCAAATAATTTAATGCAGCG;
101: GGCCGCGGACAGGAATACGAAACGTTTATACACAAAAAGTTA;
102: TCCGGATCCTTAAATACATTTGCCCAAAAAAAATGGTAAAAACAT;
103: TGCCAAGCTTTCATATATATCTACATTTGTGTAATATAATGTGG;
104: TCCGGTACCCATAATACGTAAATGTATAAATAAATATATTTAC;
105: TGGATTACTCCACTAATTTTTCAAAAAAATTGCT;
106: TGCATATCTTTATAGTATAAATGATTCTATTCAT;
107: GGCCGCGGATGTTTTTACCATTTTTTTTGGGCAAATGTATTTTA
108: TCCGAATTCAAAGAAAAAACTTTTTAAATATTTGTTATAAACTAC
16: ATGTCCATTAACATCACCATCTAATTCAACAAG;
17: GTGTTCTTTCTGATGTTCAAGAAGAAAAAGGTA;
